# Induction of miR-155 after Brain Injury Promotes Type 1 Interferon and has a Neuroprotective Effect

**DOI:** 10.3389/fnmol.2017.00228

**Published:** 2017-07-28

**Authors:** Emily B. Harrison, Katy Emanuel, Benjamin G. Lamberty, Brenda M. Morsey, Min Li, Matthew L. Kelso, Sowmya V. Yelamanchili, Howard S. Fox

**Affiliations:** ^1^Department of Pharmacology and Experimental Neuroscience, College of Medicine, University of Nebraska Medical Center Omaha, NE, United States; ^2^Department of Pharmacy Practice, College of Pharmacy, University of Nebraska Medical Center Omaha, NE, United States

**Keywords:** gene expression, injury, neurodegeneration, inflammation

## Abstract

Traumatic brain injury (TBI) produces profound and lasting neuroinflammation that has both beneficial and detrimental effects. Recent evidence has implicated microRNAs (miRNAs) in the regulation of inflammation both in the periphery and the CNS. We examined the expression of inflammation associated miRNAs in the context of TBI using a mouse controlled cortical impact (CCI) model and found increased levels of miR-21, miR-223 and miR-155 in the hippocampus after CCI. The expression of miR-155 was elevated 9-fold after CCI, an increase confirmed by *in situ* hybridization (ISH). Interestingly, expression of miR-155 was largely found in neuronal nuclei as evidenced by co-localization with DAPI in MAP2 positive neurons. In miR-155 knock out (KO) mice expression of type I interferons *IFNα* and *IFNβ*, as well as IFN regulatory factor 1 and IFN-induced chemokine *CXCL10* was decreased after TBI relative to wild type (WT) mice. Unexpectedly, miR-155 KO mice had increased levels of microglial marker Iba1 and increased neuronal degeneration as measured by fluoro-jade C (FJC) staining, suggesting a neuroprotective role for miR-155 in the context of TBI. This work demonstrates a role for miR-155 in regulation of the IFN response and neurodegeneration in the aftermath of TBI. While the presence of neuronal nuclear miRNAs has been described previously, their importance in disease states is relatively unknown. Here, we show evidence of dynamic regulation and pathological function of a nuclear miRNA in TBI.

## Introduction

Traumatic brain injury (TBI) is a leading cause of death and disability worldwide. In the US, 2.4 million people suffer a TBI each year (Coronado et al., 2012) and more than three million people are living with a long-term disability from a TBI (Zaloshnja et al., 2008). Aside from the primary mechanical injury to the brain, several secondary mechanisms contribute to morbidity and mortality; these include blood-brain barrier dysfunction, free radical production, mitochondrial dysfunction, excitotoxicity and inflammation (Finnie, 2013). Inflammation in the brain of TBI patients has been found as long as 18 years following injury and likely contributes to long-term cognitive dysfunction (Johnson et al., 2013). Therefore, targeting neuroinflammation and its downstream effects may be the key to improving TBI outcomes. It is well established that inflammatory cytokines and other inflammatory mediators can negatively affect neuronal survival (Giulian et al., 1994; Fitch et al., 1999) and function (Hauss-Wegrzyniak et al., 2002; Di Filippo et al., 2013). However, the mechanisms driving this dysfunction are still unclear. Several clinical trials aimed at reducing overall inflammation using steroids, hypothermia, hypertonic saline and eicosanoids have failed to show therapeutic efficacy (Hinson et al., 2015). These failures highlight a need for a more nuanced understanding of inflammation and it’s regulation in the context of TBI.

MicroRNAs (miRNAs) are emerging as critical regulators of immune signaling in the systemic immune system and the CNS, for detailed review please see Ksiazek-Winiarek et al. (2013), Thounaojam et al. (2013) and Cardoso et al. (2016). MiRNAs are small non-coding RNAs that are powerful post-transcriptional regulators. Mature ~22-nucleotide miRNAs target mRNA transcripts through complementary base-pairing to the 3′ UTR (Valencia-Sanchez et al., 2006). This causes target mRNA degradation or translational inhibition, allowing miRNAs to regulate the cell proteome. Through this mechanism miRNA regulate nearly all biological processes, including nervous system development, function and disease (Kosik, 2006). It is known that certain miRNAs can be induced by inflammatory stimuli. Among these are miR-155 (O’Connell et al., 2007), miR-21 (Löffler et al., 2007), miR-146 (Taganov et al., 2006), and miR-223 (Ceppi et al., 2009). What effects inflammation-associated miRNAs have on neuroinflammation in the context of TBI are unknown. Several miRNA profiling studies have shown global changes in miRNA expression after TBI in rodent models (Lei et al., 2009; Redell et al., 2009; Hu et al., 2012; Liu et al., 2014; Sun et al., 2014; Meissner et al., 2016), but there is limited knowledge about the function of these molecules in the context of brain injury. Multiple groups studying miR-21 have identified a role for this miRNA in neuroprotection and blood-brain barrier integrity after TBI (Ge et al., 2014, 2015; Han et al., 2014), indicating that miRNAs can affect TBI outcomes. Additionally, miRNAs can regulate other TBI relevant pathways, such as reactive oxidation (Zhang X. et al., 2012), excitotoxicity (Harraz et al., 2012), and even psychiatric outcomes, including major depression and suicide (Serafini et al., 2014).

We hypothesized that miRNAs known to be induced by inflammatory signaling pathways would be elevated after TBI and impact downstream inflammation and outcomes. Several miRNAs are known to be induced by inflammatory stimuli (Dai and Ahmed, 2011; O’Connell et al., 2012; Singh et al., 2013). Four of these miRNAs, miR-155, miR-21, miR-223 and miR-146 were differentially expressed in at least one miRNA profiling experiment performed in rodent models of TBI (Lei et al., 2009; Redell et al., 2009; Hu et al., 2012; Liu et al., 2014; Sun et al., 2014; Meissner et al., 2016). To study the role of inflammation-associated miRNAs in TBI, we utilized a mouse controlled cortical impact (CCI) model. In the CCI model, a pneumatically driven piston is used to impact the exposed dura creating a unilateral injury to the left parietal cortex, resulting in measurable motor and memory deficits (Saatman et al., 2006). We primarily focus on miR-155, which we found robustly increased after TBI. Using a miR-155 knock out (KO) mouse, we examined the role of miR-155 in TBI.

## Materials and Methods

### Animals

Male C57BL/6 mice were obtained from Charles River Laboratories Inc. (Wilmington, MA, USA). MiR-155 KO mice (Dagan et al., 2012), strain B6.Cg-Mir155tm1.1Rsky/J, were purchased from Jackson labs (Bar Harbor, MA, USA). C57BL/6 mice were used as wild type (WT) controls for miR-155 KO experiments. All mice were group housed in a 12 h light-dark cycle and fed *ad libitum*. All procedures and protocols were approved by the Institutional Animal Care and Use Committee of the University of Nebraska Medical Center and conducted in accordance with the National Institutes of Health Guide for the Care and Use of Laboratory Animals.

### Controlled Cortical Impact

Seven to nine week-old male mice were anesthetized using 5% inhaled isoflurane, their heads were shaved and placed in a Kopf stereotaxic head frame where they were maintained under anesthesia using 2% isoflurane. A 4-mm craniotomy was performed midway between lambda and bregma on the left side. The exposed dura was then impacted using a Precision Systems and Instrumentation TBI-0310 (Fairfax Station, VA, USA) at a speed of 3.5 m/s with a 200 ms dwell time. This procedure was similar to previous reports (Mattson and Scheff, 1994; Smith et al., 1995). Injury depths of 0.5 and 1.0 mm were used to simulate moderate and severe injuries, respectively. After injury, Surgicel (Johnson and Johnson, Dallas, TX, USA) was placed over the injury site, the removed skull was adhered back to its original place with dental cement and the wound closed with wound clips. 0.5% bupivacaine with 1:200,000 epinephrine was used as an analgesic. Naïve animals were not exposed to injury, craniotomy or anesthesia.

### Tissue Preparation for Histology

Paraffin-embedded tissues were used for Luxol fast blue, terminal deoxynucleotidyl transferase dUTP nick end labeling (TUNEL) and fluorescent *in situ* hybridization (FISH). Brains were submersion fixed in 4% PFA overnight before processing; staining was performed on 5 μm sections. Iba1 and fluoro-jade C (FJC) staining were performed on fixed-frozen tissues. Transcardial perfusion was performed on anesthetized mice with cold PBS and 2.5% sucrose followed by PBS with 2.5% sucrose and 4% PFA. Brains were submersion fixed an additional 24 h, cryoprotected in 30% sucrose, frozen with 2-methyl butane on dry ice and stored at −80°C. A 4 mm coronal section, including the injury site was sectioned into 50 μm slices and every fifth section was collected in 0.1 M phosphate buffer.

### Luxol Fast Blue Staining

After hydration to 95% ethanol, slides were stained with filtered 0.1% Luxol fast blue in a solution of 0.5% acetic acid at 60°C overnight. The next day slides were rinsed in 95% ethanol followed by distilled water then differentiated in 0.05% lithium carbonate for 1 min and 70% ethanol for 1 min. Slides were counterstained with 0.5% cresyl violet for 30 min at 60°C, rinsed in distilled H_2_O, and differentiated in 95% ethanol for 5 min. Coverslips were mounted using Cytoseal (Thermo, Waltham, MA, USA). Slide scanning was performed by the UNMC tissue sciences facility using a Ventana’s Coreo Au Slide Scanner at 40× magnification.

### Terminal Deoxynucleotidyl Transferase dUTP Nick End Labeling (TUNEL)

ApopTag Peroxidase *in situ* apoptosis detection kit (Millipore, Temecula, CA, USA) was used for TUNEL staining according to manufacturer’s directions in conjunction with TSA plus cyanine 5 kit (PerkinElmer, Waltham, MA, USA). Briefly, hydrated slides were treated with 20 μg/mL proteinase K, then terminal deoxynucleotidyl transferase (TdT) was used to transfer digoxigenin (DIG) labeled nucleotides onto fragmented DNA. The tail of DIG labeled nucleotides was then detected using an anti-DIG antibody conjugated to peroxidase. A TSA plus cyanine 5 kit (PerkinElmer, Waltham, MA, USA) was used to develop the fluorescent stain and nuclei were stained with DAPI.

### *In Situ* Hybridization

ISH was performed as described previously (Chaudhuri et al., 2013). In brief, double DIG-labeled LNA probes (Exiqon, Vedbaek) were used in conjunction with anti-DIG-POD, Fab fragments (Roche, Basel) and TSA plus cyanine 5 (PerkinElmer, Waltham, MA, USA). To reduce background, a 10 min 3% H_2_O_2_ peroxidase quenching step was added to the original protocol. MAP2 antibody (ab5392, abcam, Cambridge) was used at 1:1000 dilution; nuclei were stained with DAPI.

### Immunohistochemisty

Free-floating cryosections were incubated with 3% H_2_O_2_ to quench endogenous peroxidase. After thorough washing sections were blocked with 10% normal goat serum (NGS) +0.3% Triton-X100 in PBS. Tissues were incubated with Iba1 (Wako, Kampenhout; 1:500) in 0.3% Triton-X100 +3% NGS in PBS at 4°C overnight. ImmPRESS HRP Anti-Rabbit IgG (Vector labs, Burlingame) was used as a secondary and DAB Plus substrate system (Thermo, Waltham, MA, USA) was used for developing. Sections were then mounted on gelatin-coated slides, allowed to dry overnight, incubated in xylene for 1 min, and mounted with Cytoseal (Thermo, Waltham, MA, USA). Slides were imaged using a light microscope at 100× magnification. Images were quantified using ImageJ (Schneider et al., 2012) to calculate the percent area covered by Iba1 staining in the hippocampus and cortex.

### Fluoro-Jade C (FJC) Staining

Free-floating cryosections were mounted on gelatin coated slides and air-dried overnight. Slides were incubated in 0.06% potassium permanganate followed by 0.0001% FJC (Histochem, Jefferson, AR, USA) and 0.0001% DAPI (Sigma, St. Louis, MO, USA) in 1% acetic acid. Tissue was then dried in a 60°C oven and incubated in xylene for 1 min, then coverslips were mounted with DPX (Sigma, St. Louis, MO, USA). For each brain, three slices between bregma −2.5 and −1.5 were imaged for quantification. FJC positive cells were counted using ImageJ (Schneider et al., 2012) by a blinded observer. The average number of fluoro-jade positive cells per slice is reported.

### RNA Isolation, cDNA Synthesis and Real Time PCR

Tissue was isolated from the hippocampus and RNA was extracted using TRIzol (Life Technologies, Carlsbad, CA, USA) according to manufacturer’s instructions. TaqMan miRNA and Gene Expression Assays (Life Technologies, Carlsbad, CA, USA) were used for cDNA synthesis and real time PCR according to manufacturer’s instructions. Small nuclear RNA U6 (*U6*) was used as a control for miRNA studies and glyceraldehyde 3-phosphate dehydrogenase (*GAPDH*) was used as a control for mRNA studies. Delta-delta Ct method was used to calculate fold change (2^−^^((Ct_miRNA_ − Ct_U6_)_exp_ − (Ct_miRNA_ − Ct_U6_)_control_)^) or (2^−^^((Ct_mRNA_ − Ct_GAPDH_)_exp_ − (Ct_mRNA_ − Ct_GAPDH_)_control_)^). Statistical significance was determined using ΔCt values (Ct_miRNA_ − Ct_U6_) or (Ct_mRNA_ − Ct_GAPDH_).

### Protein Analysis

Protein was isolated from ipsilateral hippocampal tissue using TRIzol (Life Technologies, Carlsbad, CA, USA) according to manufacturer’s instructions. Equal amounts of protein samples were separated by SDS-PAGE and transferred to a nitrocellulose membrane. The membrane was blocked in Superblock (Pierce, Waltham, MA, USA) and incubated overnight at 4°C with primary antibodies. After washing, membrane was incubated with infrared secondary antibodies (LI-COR, Lincoln, NE, USA) for 1 h at room temperature. Imaging and densitometry was performed with an Odyssey Imaging System (LI-COR, Lincoln, NE, USA). The following antibodies and concentrations were used for Western blotting: suppressor of cytokine signaling 1 (SOCS1; 1:1000, 38-5200, Thermo, Waltham, MA, USA), caspase 3 (1:1000, 9662, Cell Signaling, Danvers, MA, USA), beta-actin (AM1829B, 1:20,000, Abgent, San Diego, CA, USA). For statistical analysis, each sample was normalized to beta-actin.

### Statistics

For comparisons between two groups an unpaired two-tailed student’s *T*-test was used to calculate significance. For comparisons between multiple groups two-way ANOVAs followed by Bonferroni *post hoc* tests were employed. For testing significance of multiple parameters, such as the expression of several genes, between two groups a false discovery rate calculation was used. All statistical tests were performed with GraphPad Prism (La Jolla, CA, USA). *P* < 0.05 was considered significant. For data represented in graphs, the mean ± SEM is shown. A minimum of three biological replicates was used for all experiments.

## Results

### CCI Increased Expression of Inflammation-Associated miRNAs in the Hippocampus

**Figure 1:**
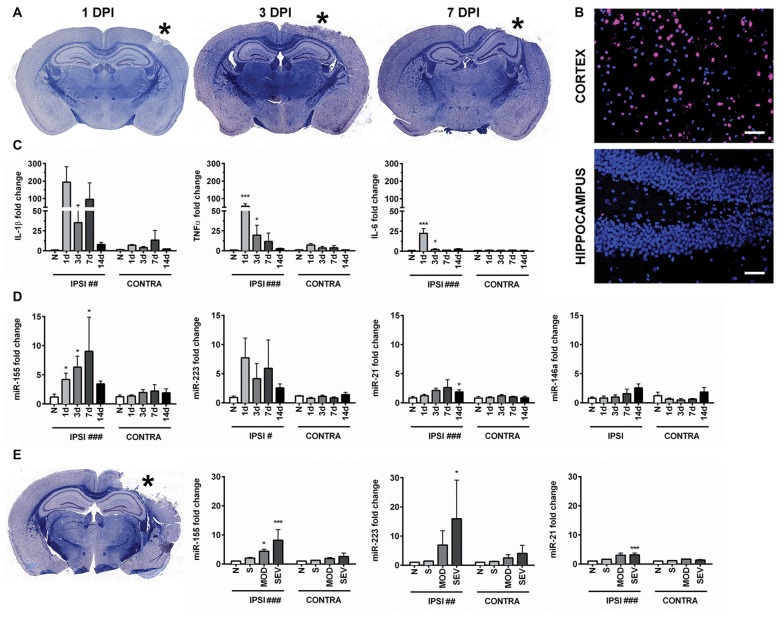
Controlled cortical impact (CCI) increases inflammation-associated microRNAs (miRNAs) in the hippocampus. **(A)** Anatomical characterization of moderate CCI 1, 3, or 7 days post injury (DPI), myelin (Blue) and Nissl (Purple). **(B)** Staining for apoptotic cells using terminal deoxynucleotidyl transferase dUTP nick end labeling (TUNEL; magenta), nucleus (blue). Images show ipsilateral hemisphere 1 day after moderate CCI in the hippocampus and the lesion boundary of the cortex. Scale bars = 50 μM. **(C)** Levels of inflammatory cytokines and **(D)** inflammation-associated miRNAs in the ipsilateral (IPSI) and contralateral (CONTRA) hippocampus 1, 3, 7 and 14 days after CCI by qPCR at 0.5-mm injury depth and in naïve animals (*n* = 3). **(E)** Anatomical characterization of severe CCI 3 DPI (left) and levels of miRNA in the hippocampus (right) were measured 3 days after injury by qPCR in naïve (*n* = 4), sham (*n* = 4), moderate CCI (*n* = 3), and severe CCI mice (*n* = 4). Data are expressed as fold change relative to naïve mice. The mean ± SEM are shown. ^#^*P* < 0.05; ^##^*P* < 0.01; ^###^*P* < 0.001 by two way ANOVA. *P < 0.05; ***P < 0.001 by Bonferroni *post hoc* testing relative to the contralateral hemisphere.

CCI produced a cortical lesion, and white matter damage to the underlying corpus callosum and alveus was evident later time points (Figure 1A). Given that apoptosis can occur in TBI and may be influenced by the miRNAs of interest, we examined the level of apoptosis in the cortex and hippocampus. Importantly, apoptosis in the hippocampus is limited compared to the cortical lesion boundary, which shows high levels of TUNEL staining (Figure 1B). To confirm that inflammatory signaling was present in the hippocampus, we measured the expression of pro-inflammatory cytokines *IL-1β*, *TNFα* and *IL-6* in the hippocampus 1, 3, 7 and 14 days after injury by qPCR. As expected based on previous reports, expression of pro-inflammatory cytokines was increased acutely after CCI in the injured (ipsilateral) compared to the uninjured (contralateral) hippocampus (Figure 1C). This indicated that the hippocampus was a suitable region for studying regulation of inflammation by miRNAs while limiting the confounding role of apoptosis.

Expression of miR-155 (UUAAUGCUAAUUGUGAUAGGGGU), miR-21(UAGCUUAUCAGACUGAUGUUGA), miR-223 (UGUCAGUUUGUCAAAUACCCCA) and miR-146 (UGAGAACUGAAUUCCAUGGGUU) was examined in hippocampi of the injured (ipsilateral) and the uninjured (contralateral) hemisphere from the same animals at 1, 3, 7 and 14 days after moderate CCI and in naïve controls (Figure 1D). In naïve animals, there was no difference in miRNA expression between hemispheres. CCI significantly increased expression of miR-155, miR-21 and miR-223 in the ipsilateral compared to the contralateral hippocampus by two-way ANOVA. Bonferroni *post hoc* analysis showed that the difference in miR-155 reached statistical significance at day 1, 3 and 7 whereas, expression of miR-21 was significantly increased at 14 days after CCI (*P* = 0.035), but not at earlier time points, day 1, 3 and 7. The *post hoc* test did not reveal significance for miR-223 expression at these time points. Expression of miR-146 was not altered by CCI at the time points examined by two way ANOVA (*P* = 0.16). Increasing the injury severity from moderate to severe, 0.5 mm and 1.0 mm depth respectively, revealed significantly increased expression of miR-155, miR-21 and miR-223 at 3 days post-injury (DPI) as determined by two-way ANOVA. Bonferroni *post hoc* analysis showed that miR-155, miR-223 and miR-21 were all elevated in the ipsilateral as compared to the contralateral hemisphere 3 days after severe CCI, whereas only miR-155 was also elevated after moderate CCI (Figure 1E). In summary, inflammation-associated miRNAs are elevated in the ipsilateral hippocampus after TBI and in general showed a more prolonged increase compared to the expression of cytokines *IL-1β*, *TNFα* and *IL-6*, which peaked 1 day after injury. Of the miRNAs examined, miR-155 alone was significant at all time points measured up to 7 days after moderate injury. Considering the strong induction of miR-155, the function of miR-155 in TBI was further investigated.

**Figure 2:**
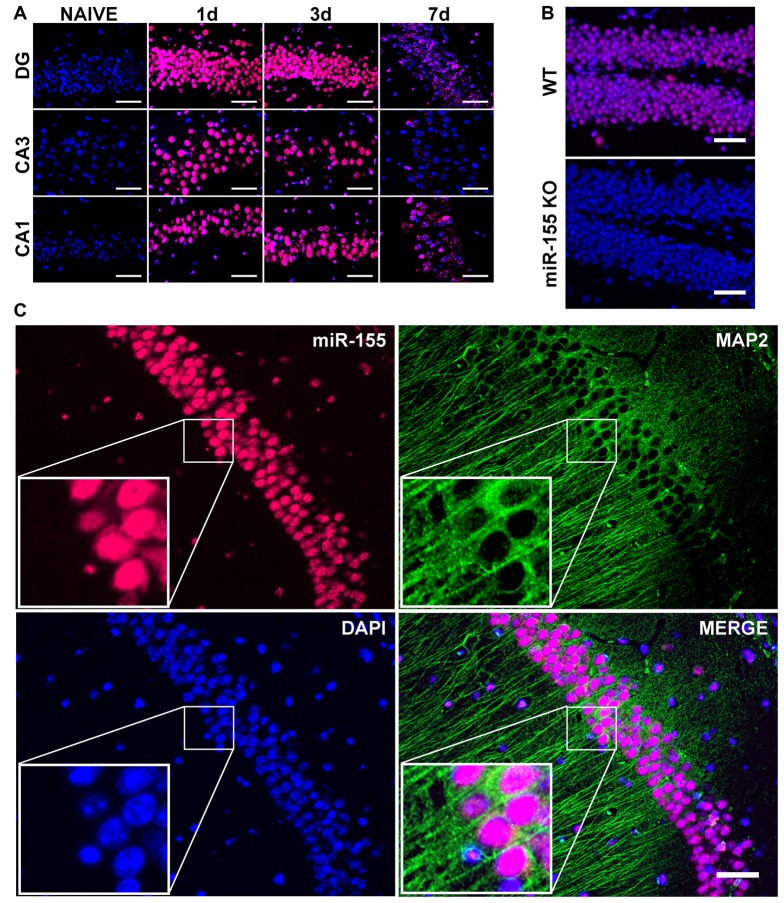
Nuclear, neuronal localization of miR-155 in the hippocampus after CCI.** (A)** Fluorescence *in situ* hybridization (FISH) for miR-155 1, 3 and 7 days after moderate CCI and in naïve mice. Dentate gyrus (DG), CA1 and CA3 of the ipsilateral hippocampus are shown. **(B)** miR-155 expression in the ipsilateral DG of wild type (WT) and miR-155 knock out (KO) mice 3 days after severe CCI. **(C)** FISH and Co-IHC was performed for neuronal marker MAP2 (green), nuclei (blue), miR-155 (magenta). Scale bars = 50 μM.

To examine the cell type-specific localization of miR-155 in the injured brain, we performed FISH. Examining miR-155 by FISH revealed prolonged elevation of miR-155 at 1, 3 and 7 days after moderate CCI as well as 3 days after severe CCI in the hippocampus, similar to that observed by qPCR (Figures 2A,B). Expression of miR-155 was also observed in the boundary zone of the cortex (Supplementary Figure S1A). Importantly, no fluorescent signal was detected in miR-155 knockout (KO) mice, confirming FISH specificity (Figure 2B). Interestingly, expression of miR-155 was largely nuclear and present at high levels in the laminar layers of the hippocampus, structures composed primarily of neurons. Co-staining for miR-155 and neuronal marker MAP2 confirmed that miR-155 was expressed at high levels in neuronal nuclei in both the hippocampus and cortex (Figure 2C, Supplementary Figure S1B). Together with qPCR data, FISH clearly shows elevation of miR-155 after CCI.

### Interferon Signaling Is Dampened in miR-155 KO Mice

**Figure 3:**
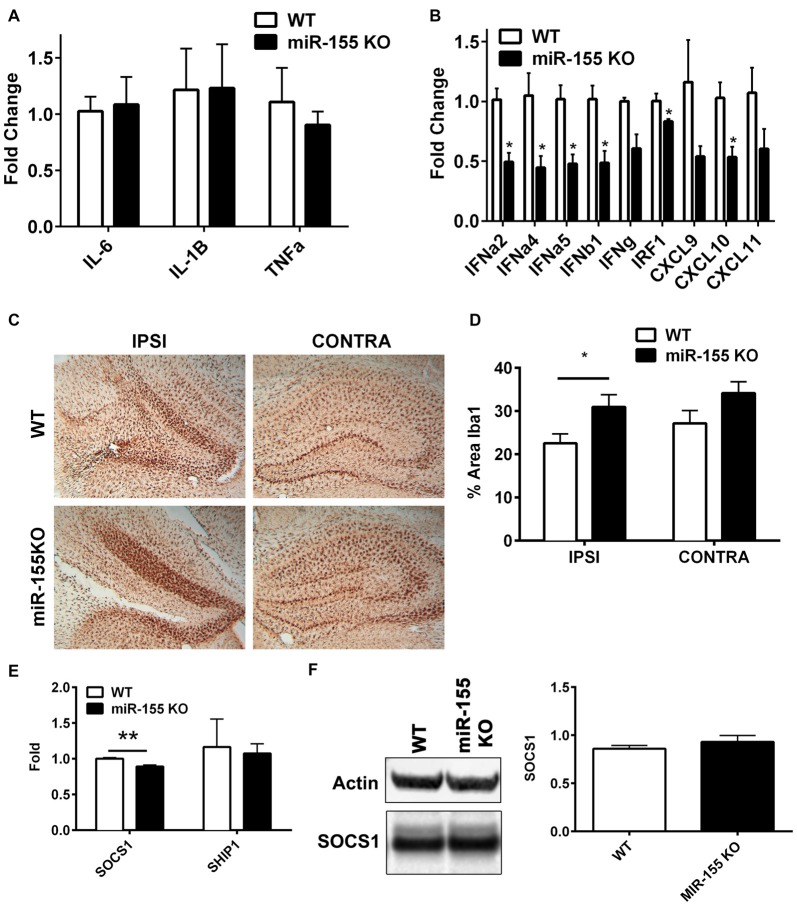
miR-155 KO mice have a dampened hippocampal interferon (IFN) response to CCI. **(A)** Levels of pro-inflammatory cytokines *IL-6*, *TNFα* and *IL1β*, and **(B)** IFNs, IFN regulatory factor 1 (*IRF1*) and IFN-induced chemokines in the ipsilateral hippocampus were measured 3 days after severe CCI by qPCR in miR-155 KO and WT mice. Data are expressed as fold change relative to WT, the mean ± SEM are shown (*n* = 4). *FDR < 0.05. **(C)** Brains from WT and miR-155 KO mice were stained for Iba1 3 days after severe CCI. Representative images are shown. **(D)** Quantification of Iba1 staining of WT (*n* = 5) and miR-155 KO (*n* = 6) ipsilateral hippocampi. The mean ± SEM are shown. *P = 0.046 by Student’s *T*-test. **(E)** Levels of suppressor of *SOCS1* and *SHIP1* mRNA and **(F)** SOCS1 protein in the ipsilateral and contralateral hippocampus were measured 3 days after severe CCI in WT (*n* = 4) and KO (*n* = 4) mice by qPCR and Western blot respectively. The mean ± SEM from are shown. **P = 0.0072 by Student’s *T*-test.

In order to understand the function of miR-155 in regulating the immune response in TBI, we subjected miR-155 KOs and WT mice to CCI. Three days after CCI, RNA was isolated from ipsilateral hippocampi and expression of cytokines and chemokines were measured by qPCR. Unexpectedly, there were no differences in the expression of *IL-6*, *IL-1β* or *TNFα* (Figure 3A), which are regulated by miR-155 in other systems. However, miR-155 KO mice had decreased expression of 6/9 IFN related genes measured (FDR < 0.05), including *IFNα2*, *IFNα4*, *IFNα5* and *IFNβ1*, as well as IFN regulatory factor 1 (*IRF1*) and IFN induced chemokine *CXCL10* (Figure 3B). Overall, we found a reduction of the IFN response in miR-155 KO mice after TBI without changes in other TBI induced inflammatory cytokines.

To examine the importance of miR-155 in microglial activation in the context of TBI, we examined the levels of microglial marker Iba1 in hippocampal slices 3 days after CCI in miR-155 KO and WT mice. In miR-155 KO mice we observed an increase rather than the expected decrease in Iba1 staining in the ipsilateral hippocampus compared to control mice (*P* = 0.046 by Student’s *T*-test; Figures 3C,D). These data suggest that miR-155 is not essential for the activation of microglia after TBI and implies that a dampened microglial response is not responsible for the reduction in IFN signaling observed in miR-155 KO mice.

We hypothesized that previously validated miR-155 targets *SOCS1* and *SHIP1* mediated miR-155 regulation of IFN signaling in the context of TBI. However, compared to the expression found in WT mice, no increase in SOCS1 was observed in miR-155 KO mice at the mRNA or protein level after TBI (Figures 3E,F). In fact there was a significant decrease in *SOCS1* mRNA expression in miR-155 KO mice (*P* = 0.007 by student’s *T-test)*. Therefore, miR-155 regulation of IFN signaling in TBI is likely independent of SOCS1. We also tested *SHIP1*, a second target of miR-155 known to regulate IFN signaling and found no significant change in mRNA expression by qPCR. In summary, miR-155 KO mice show a decreased expression of type 1 IFNs and IFN regulated genes without evident decreases in microglial activation or increased *SOCS1* or *SHIP1* expression.

### Increased Neurodegeneration in miR-155 KO Mice

**Figure 4:**
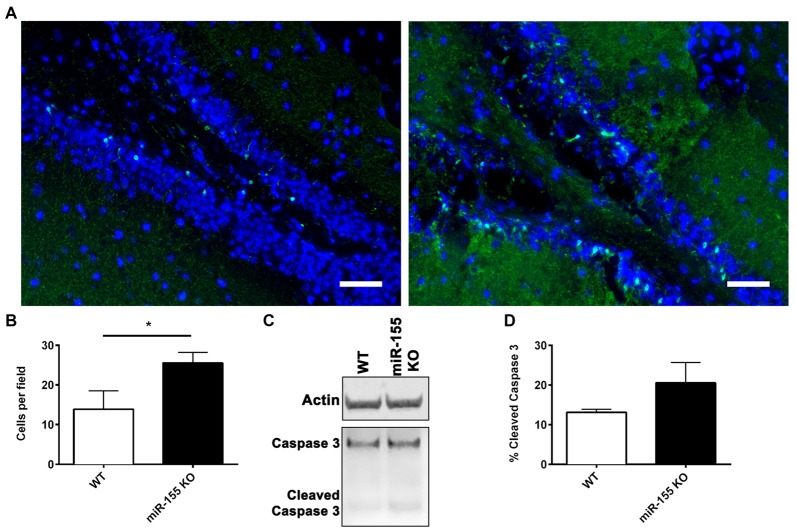
Increased hippocampal neurodegeneration in miR-155 KO mice after CCI. Degenerating neurons were stained with fluoro-jade C (FJC; green) and DAPI (blue) 3 days after 1.0-mm CCI in the brains of WT (*n* = 5) and miR-155 KO (*n* = 6) mice. **(A)** Representative images from the ipsilateral DG. **(B)** Quantification of FJC positive cells in the DG. The mean ± SEM is shown. *P < 0.05 by Student’s *T*-test. **(C)** Representative images of full-length and cleaved caspase 3 and beta-actin (Actin) analyzed by Western blot in WT and miR-155 KO ipsilateral hippocampi 3 days after 1.0-mm CCI. **(D)** Quantification of the percentage of cleaved Caspase 3 relative to full-length Caspase 3. The mean ± SEM are shown, (*n* = 4).

**Figure 5:**
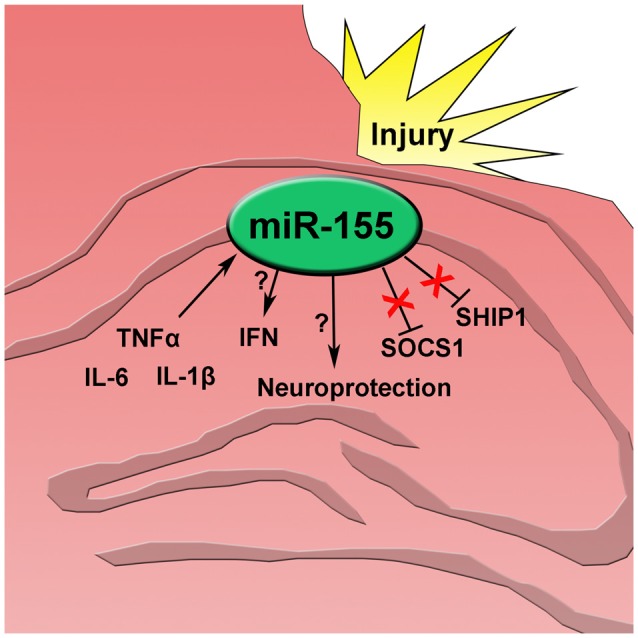
After traumatic brain injury inflammatory cytokines tumor necrosis factor alpha (TNFα), interleukin 6 (IL-6), and interleukin 1 beta (IL-1β) increase and induce miR-155 expression in the injured hippocampus. miR-155 promotes type 1 interferon (IFN) though an unknown mechanism independent of SOCS1 and SHIP1. Also, expression of miR-155 has a neuroprotective effect after injury.

Since miR-155 was highly increased in neuronal nuclei, the effect of miR-155 ablation on neuronal degeneration and apoptosis in the hippocampus was measured. Fluoro-jade C (FJC) staining for degenerating neurons showed evidence of neurodegeneration both in the cortex and hippocampus. In the hippocampus FJC positive cells were found predominately in the Dentate gyrus (DG). The DG in miR-155 KO mice had significantly more FJC positive cells relative to controls (*P* = 0.046 by Student’s *T*-test; Figures 4A,B), indicating that miR-155 KO mice had increased hippocampal neurodegeneration. Consistent with TUNEL staining of the hippocampus, there were low levels of caspase 3 cleavage in both groups, 10% and 20% for WT miR-155 KO mice respectively, a difference that was not significant (*P* = 0.20 by Student’s *T*-test; Figures 4C,D). Overall we found increased hippocampal neurodegeneration in miR-155 KO mice, without significant evidence of increased apoptosis. Figure 5 graphically summarizes our main findings on miR-155 in TBI.

## Discussion

Neuroinflammation has a complex role in TBI playing both beneficial and detrimental roles (Finnie, 2013). Understanding the regulation and downstream effects of inflammation in the context of brain injury will help unravel this seeming duplicitous process. The expression of inflammation-associated miRNAs was examined in the CCI mouse model of TBI. Inflammation-associated miRNAs miR-155, miR-21 and miR-223 were increased in the hippocampus after CCI. Of the miRNAs examined, miR-155 showed the most consistent upregulation, up to 9-fold and extending to 7 days after CCI. Using a miR-155 KO mouse, we identified a role for miR-155 in promoting the expression of IFN genes, including *IFNα2*, *IFNα4*, *IFNα5* and *FNβ1*, as well as *IRF1* and IFN induced chemokine *CXCL10*. Surprisingly, we found that miR-155 was highly expressed in neuronal nuclei suggesting potential non-canonical functions of this miRNA in TBI. Additionally, we found that miR-155 KO mice had increased neurodegeneration and microgliosis, indicating a neuroprotective role for miR-155 in neuronal injury. This work confirms that miR-155 regulates neuroinflammation and also provides evidence that miR-155 plays a role in the neuronal response to TBI.

Increasing evidence points to important functions for inflammation-associated miRNAs in neurological disease (Buller et al., 2010; Yelamanchili et al., 2010; Harraz et al., 2012; Zhang L. et al., 2012; Ge et al., 2014). Among the miRNAs associated with neuroinflammation, miR-155 is the best characterized, having well-documented roles in several disease models. Inhibiting or reducing miR-155 improved outcomes in several neural disease models involving neuroinflammation, such as Parkinson’s disease (Thome et al., 2016), Alzheimer’s disease (Guedes et al., 2014), amyotropic lateral sclerosis (Koval et al., 2013; Parisi et al., 2013; Butovsky et al., 2015b), stroke (Eisenhardt et al., 2015), and alcohol induced inflammation (Lippai et al., 2013). Intriguingly, miR-155 mice are also highly susceptible to HSV encephalitis (Bhela et al., 2014). Our data indicate that TBI produces a robust increase in miR-155 at 1, 3 and 7 DPI with a return to baseline by 14 days. Increased expression after TBI was confirmed by ISH. Expression of miR-155 is induced by TNFα (O’Connell et al., 2007) and IL-1β (Stanczyk et al., 2008). TNFα and IL-1β mRNA expression increased dramatically after TBI, 200 fold and 50 fold from naive respectively. Therefore, we propose that TNFα and IL-1β signaling leads to miR-155 expression after TBI.

Surprisingly, miR-155 was detected in the nucleus of cells. Nuclear miRNAs have been shown to mediate gene expression changes in non-canonical ways such as alternative splicing and chromatin modification in addition to post-transcriptional inhibition (Roberts, 2014). Some additional functions include degradation of long non-coding RNAs, disruption of pri-miRNA processing, and transcriptional activation (Rasko and Wong, 2017). Differential CLIP-Seq of miR-155 KO cells by Loeb et al. (2012) revealed many non-canonical miR-155 binding sites, including sites in small nucleolar RNAs (snoRNAs). Nuclear localization of miRNAs has been reported in neurons, but what role nuclear-neuronal miRNAs play in development and disease is largely unknown (Khudayberdiev et al., 2013). Here we describe a nuclear-neuronal miRNA that was not only induced in a neurological condition, but also played a functional role in disease processes. This discovery paves the way for the study of nuclear-neuronal miRNA in neurological disease.

In the brains of miR-155 KO mice the type 1 IFN response was decreased. This is consistent with the findings of several other groups studying the role of miR-155 in the immune system (Zhou et al., 2010). IFNβ is increased after both murine and human TBI (Karve et al., 2016). Toll-like receptors and other pattern recognition receptors (PRRs) are responsible for stimulating type 1 IFNs in response to viral RNA (Honda and Taniguchi, 2006). In TBI, IFN induction could be mediated by a variety of damage associated molecular patterns (DAMPS) released upon injury (Gesuete et al., 2014). For example we have shown that miRNA containing TLR7 recognition motifs are secreted in extracellular vesicles after TBI (Harrison et al., 2016). Karve et al. (2016) showed that deletion of the IFNA1 receptor improved outcomes in murine TBI and that these effects were mediated by hematopoietic cells. In addition to hematopoietic cells, neurons can both express and respond to type 1 IFNs (Delhaye et al., 2006; Rosato and Leib, 2015). Therefore, it is possible that in the context of TBI miR-155 promotes IFN expression in neurons.

Despite evidence that miR-155 promotes microglial activation *in vitro* (Cardoso et al., 2012), we did not see a reduction in microgliosis in miR-155 KO after CCI mice as measured by Iba1 staining density. This study only provides a limited characterization of microglial activation and it is possible that by using markers more specific to microglial activation or examining a more extended time course that alteration in microglial activation may be present in miR-155 KO mice after TBI. Therefore, we cannot rule out the importance of miR-155 for microglial activation, but we argue that the role for miR-155 in response to CNS injury extends beyond glial activation. Specifically, expression of miR-155 in MAP2 expressing neurons suggests that this miRNA may have an equally important role in neurons as in glia.

There is evidence that miR-155 contributes to neuroinflammation by reducing SOCS1 protein levels in microglia and astrocytes (Cardoso et al., 2012; Butovsky et al., 2015a). SOCS1 is a negative regulator of IFN signaling and a validated target of miR-155 (Wang et al., 2010) that functions to inhibit STAT1, a key transcription factor for IFN signaling (Nakagawa et al., 2002). In miR-155 KO mice there was decreased expression of IFN and IFN response genes after TBI, but no corresponding increase in SOCS1 at the protein or mRNA level. Instead, we found a slight decrease in *SOCS1* mRNA. These data suggest that in the context of TBI, SOCS1 does not mediate potentiation of the IFN response by miR-155. We also found no change in expression of *SHIP1*, a validated target of miR-155 also known to inhibit IFN expression (O’Connell et al., 2009; Gabhann et al., 2010; Figure 5). This does not preclude the possibility that other canonical mRNA targets are involved. However, the nuclear localization of miR-155 also opens the possibility for non-canonical regulation of gene expression, which should also be explored.

Importantly, we found that in addition to regulating neuroinflammation, miR-155 also influenced neurodegeneration as measured by fluoro-jade C (FJC) staining. It is possible that autocrine or paracrine IFN signaling is neuroprotective and inhibition of IFNs and IFN-related genes in miR-155 KO mice promotes neurodegeration. Though the mechanisms are incompletely understood, IFNβ is a main line therapy for multiple sclerosis where it has been shown to have both anti-inflammatory and neuroprotective effects (Kieseier, 2011). Alternatively, the neuroprotective effects of miR-155 may be independent of IFN signaling and relate to either canonical or non-canonical regulation of gene expression within neurons. Further *in vitro* studies on miR-155 in neurons would help elucidate the mechanisms by which this miRNA regulates neurodegeneration.

In the brain, miRNAs regulate neuronal plasticity and neuroprotection (Kosik, 2006). Several of these processes have relevance to TBI, where both neurogenesis and regeneration are known to occur (Nudo, 2011). In addition, miRs have been associated with psychiatric disorders found in TBI survivors, such as major depression (Serafini et al., 2014). For example, increased miR-185 was found in the brains of suicide completers and linked with decreased TrkB-T1 (Maussion et al., 2012). We have shown that miR-155 is neuroprotective in the acute period after TBI, but further exploration of this and other miRNA in the chronic phase of TBI is warranted.

This study has several limitations. One important limitation is the focus on pathology and gene expression in the hippocampus. We show by qPCR that miR-155 is increased in the hippocampus after TBI. We also found increased hippocampal and cortical expression of miR-155 by FISH. The expression and function of miR-155 in other regions was not examined, but may be relevant to the overall importance of miR-155 in TBI. This work focused on a specific subset of miRNAs, but there is a growing list of other non-coding RNAs. These include long non-coding RNAs (lncRNAs), endogenous siRNAs (esiRNA) and piwi-interacting RNAs (piRNAs). Epigenetic regulation by non-coding RNAs likely plays critical roles in the response to CNS injury through chromatin remodeling, transcriptional activation and gene silencing (Esteller, 2011).

These findings clearly show a functional role for miR-155 in the mouse CCI model of TBI, although additional studies in other models of TBI as well as in human patients are necessary to show miR-155 is clinically relevant. Understanding the complex cell signaling, gene expression and epigenetic mechanisms that occur in the wake of TBI is important for improving therapeutic approaches. This work highlights changes in miRNAs, large-scale regulators of protein expression. The elevation of these miRNAs suggests that they play a role in the pathophysiology of TBI. These data show that miR-155 can affect TBI pathophysiology, but whether miR-155 expression can improve or worsen disease outcomes is a critical question that remains to be addressed.

### Summary

We have identified miR-155 as a TBI induced miRNA that promotes the type 1 IFN response. In the context of TBI, miR-155 is strongly expressed in neuronal nuclei, suggesting that it may regulate gene expression through non-canonical mechanisms. Increased neurodegeneration in miR-155 KO mice points to a neuroprotective role for miR-155 in TBI.

## Author Contributions

SVY, MLK, EBH and HSF conceived and designed the project. EBH, KE, BGL, BMM and ML collected and analyzed the data. EBH, ML, BGL, KE and SVY interpreted the data. EBH and HSF wrote the manuscript. SVY, MLK, HSF, ML, BGL, KE and BMM all contributed to the editing of the manuscript.

## Conflict of Interest Statement

The authors declare that the research was conducted in the absence of any commercial or financial relationships that could be construed as a potential conflict of interest.

## Supplementary Material

FIGURE S1 Nuclear, neuronal localization of miR-155 in the cortex after controlled cortical impact (CCI). **(A)** Fluorescence *in situ* hybridization (FISH) for miR-155 1, 3 and 7 days after moderate CCI and in naïve mice. Images show the injury lesion boundary in the cortex. **(B)** FISH and Co-IHC was performed for neuronal marker MAP2 (green), nuclei (blue), miR-155 (magenta). Scale bars = 50 μM.

## Abbreviations

CCI: controlled cortical impact
IFN: Interferon
miRNA: microRNA
TBI: traumatic brain injury.
