# Supplementary figures and images for: Induction of miR-155 after Brain Injury Promotes Type 1 Interferon and has a Neuroprotective Effect
Source: Front Mol Neurosci. 2017 Jul 28;10:228. doi: 10.3389/fnmol.2017.00228 (PMC5532436; doi:10.3389/fnmol.2017.00228)

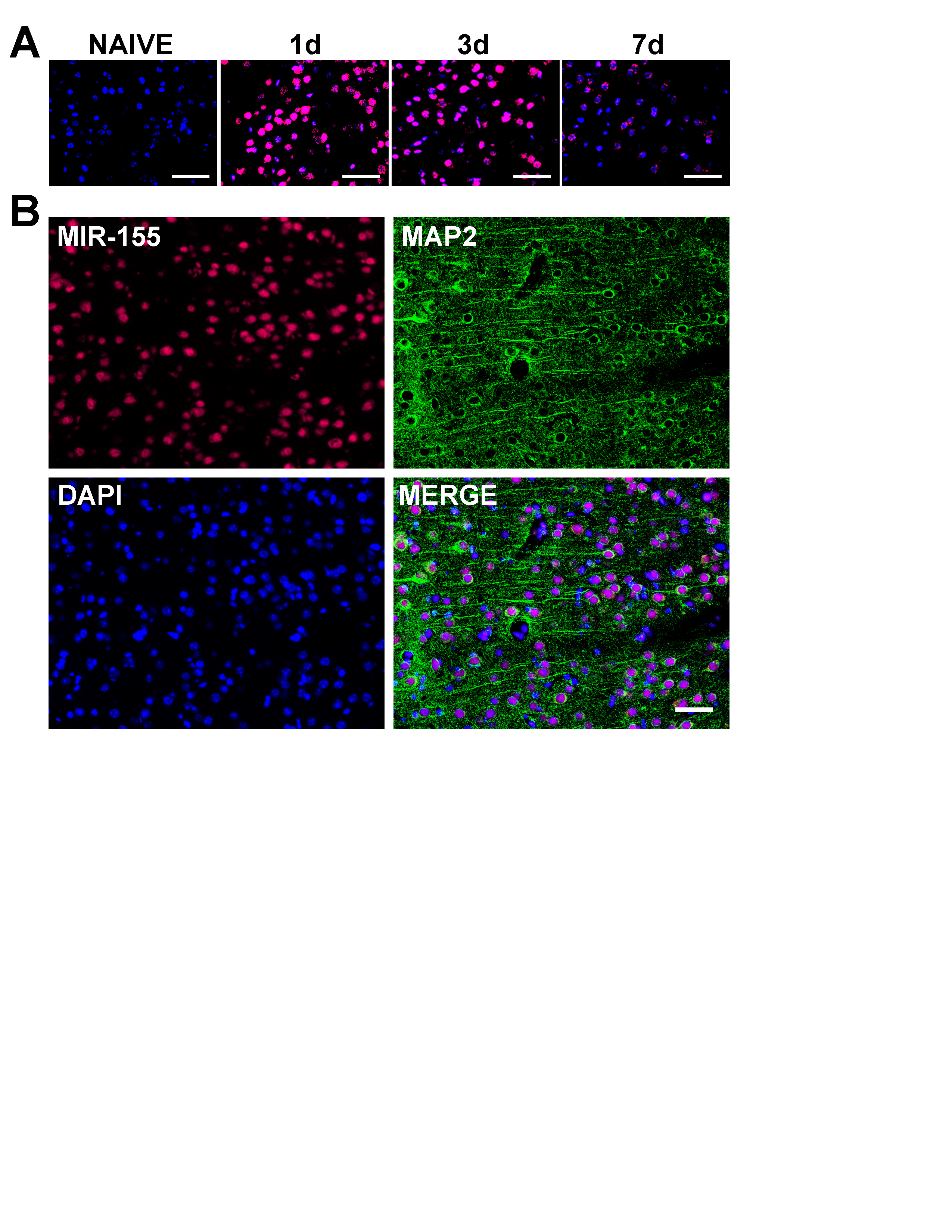

Supplement: FIGURE S1 — Nuclear, neuronal localization of miR-155 in the cortex after controlled cortical impact (CCI). (A) Fluorescence in situ hybridization (FISH) for miR-155 1, 3 and 7 days after moderate CCI and in naïve mice. Images show the injury lesion boundary in the cortex. (B) FISH and Co-IHC was performed for neuronal marker MAP2 (green), nuclei (blue), miR-155 (magenta). Scale bars = 50 μM. [file Image_1.tif]
